# Outcomes and Complications of Bronchial Angioembolization in Patients with Massive Hemoptysis

**Published:** 2019-04

**Authors:** Seyed Reza Seyyedi, Parham Sadeghipour, Makan Sadr, Omid Shafe, Jamal Moosavi, Oldooz Aloosh, Atefeh Abedini, Babak Sharif-Kashani

**Affiliations:** 1 Lung Transplantation Research Center, Department of Cardiology, National Research Institute of Tuberculosis and Lung Diseases, Shahid Beheshti University of Medical Sciences, Tehran, Iran; 2 Cardiovascular Intervention Research Center, Rajaie Cardiovascular Medical and Research Center, Iran University of Medical Sciences, Tehran, Iran; 3 Virology Research Center, National Research Institute of Tuberculosis and Lung Diseases, Shahid Beheshti University of Medical Sciences, Tehran, Iran

**Keywords:** Angioembolization, Bronchial artery, Hemoptysis

## Abstract

**Background:**

Massive hemoptysis is a potentially life threatening medical condition and one major problem for both patients and physicians. Choosing the appropriate treatment for the patients is crucial in order to decrease the complications and increase the success rate. Hence, in this study the outcomes and complications of bronchial angioembolization (BAE) were determined in patients with massive hemoptysis.

**Materials and Methods:**

In this prospective cohort, 189 consecutive patients with moderate and severe hemoptysis who had referred to two large cardiovascular centers were enrolled. The Chest X Ray, CT Scan, Fiberoptic Bronchoscopy, Selective and Nonselective Bronchial Angiography were performed in patients. The outcomes with 20-month follow-up were compared.

**Results:**

The immediate success rate was 97.3%. In 79.7% there were no complications. Temporary chest pain, subintimal dissection, temporary dysphagia, and pancreatitis were seen in 12.3, 2.4, 5.1, and 0.5%, respectively, without any major complication. The in-hospital mortality rate was 1.1% and mortality during 20-month follow-up was 9.6%, and recurrence rate was 28.3% on total.

**Conclusion:**

Our case series showed that BAE is a safe and effective method in treating patients with hemoptysis. Compared to surgery, the procedure is faster and less invasive and might be used both as first line or bridging therapy. Importantly, no major complications have been detected.

## INTRODUCTION

Hemoptysis is the coughing up of blood or bloody sputum from respiratory tract originating from below the vocal cord (1). It is a concerning issue for both patients and physicians because of life threatening situation and serious etiologies such as malignancy (2). While mild hemoptysis is discontinued spontaneously in vast majority of cases, massive hemoptysis may be a life-threatening problem (3). Massive hemoptysis is defined as coughing up of massive bloody sputum (more than 240 to 300 ml) in 24 hours. It is a critical condition with case fatality rate of nearly fifty percent (4, 5).

The etiologies for hemoptysis are currently changed from tuberculosis, bronchitis, and bronchiectasis to malignancy (6–8). Treatments are also different according to the background etiologies (9). The surgical and endovascular therapeutic modalities are main treatment options with success rate of 85 to 100 percent in endovascular method and relatively lower in surgical modality with recurrence rate of 10 to 33 percent (10). First described in 1973, angioembolization is a novel therapeutic option with fewer invasions versus surgical methods; if the success rate is established it may be the therapeutic choice due to lower rate of adverse effects (10, 11).

Despite multiple studies worldwide, there is no large extent clinical study among Iranian subjects. In the present study, we have published our short-term and 20-month follow-up outcome of patients presenting in two large cardiovascular centers in Iran.

## MATERIALS AND METHODS

From August 2016 to April 2019, 189 consecutive patients with massive hemoptysis who referred to two large cardiovascular centers- Masih Daneshvari Hospital and Rajaie Cardiovascular Medical and Research Center, were enrolled in this prospective study. The study was approved by the ethical committee.

Inclusion criteria were patients with massive hemoptysis, not candidate for surgical therapy. The exclusion criteria were cases with spinal artery originating from the culprit bronchial artery, while passage of microcatheter beyond the side branch was impossible.

### Bronchial Angioembolization (BAE)

In non-emergency situation, before the embolization, culprit vessel is routinely identified in the CT angiography. In addition, the best working view of each bronchial artery is also determined. In contrary, in emergency situation patient’s condition do not usually allow for preprocedural imaging. In this group of patients saving airways is of utmost importance and thus anesthesiologist consultation is mandatory.

The procedure is usually performed via a femoral access. Heparin is contraindicated during the procedure and consequently constant flushing is mandatory during the procedure. With the help of a descending thoracic aortogram, the exact anatomical location of bronchial arteries was detected. Next, selective bronchial artery angiography was performed. In our experience, selective engagement might be facilitated with Judkins left catheter, Tiger catheter and Cobra catheter. Abnormal angiographic findings such as vessel enlargement and tortuosity, parenchymal blushing, shunting to PA or vein, aneurysm & contrast extravasation confirm the culprit vessel. Cannulation of the culprit vessel was performed with the help of a 0.014– 300 cm length wire followed by the advancement of microcatheter. The advantage of using microcatheter is to embolize in a more selective way thus, decreasing the potential complication. Polyvinyl alcohol (PVA) and EmboSphere (Merit medical, South Jordan, USA) were used for BAE. Microparticles sizing ranged from 500 μm to 900 μm. All patients were followed for 24 months.

### Statistical analysis

All the statistical analyses were performed using SPSS software, version 23.0, (SPSS, Inc, Chicago, IL, USA). All the tests were 2-tailed, and the differences were reported as significant if the *P* value was less than 0.05. The Kolmogorov–Smirnov test was applied to evaluate the normal distribution of the data. The normally distributed continuous data, expressed as the mean ± the standard deviation (SD), were analyzed using the Student *t*-test, the χ^2^ test, or the Fisher exact test. The continuous variables were compared between the 2 groups and in each group before and after the intervention by using the independent *t*-test and the paired sample *t*-test, correspondingly.

## RESULTS

**Figure 1.**
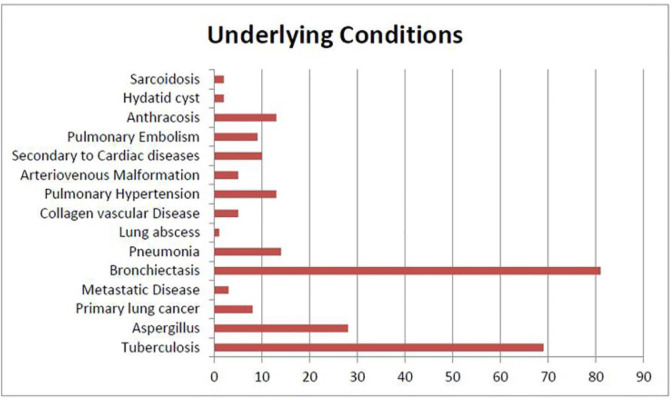
Etiology for hemoptysis

There were 109 male subjects (58.3%). The immediate success rate was 97.3%. In 79.7% of patients there were no complications. Temporary chest pain, subintimal dissection, temporary dysphagia and pancreatitis were seen in 12.3, 2.4, 5.1 and 0.5%, respectively without any major complication. In hospital mortality rate was 1.1%, mortality during 20-month follow-up was 9.6% and recurrence rate was 28.3% totally. The etiologies of hemoptysis and their prevalence have been depicted in figure 1. Bronchiectasis followed by Tuberculosis was the most common causes.

**Figure 2.**
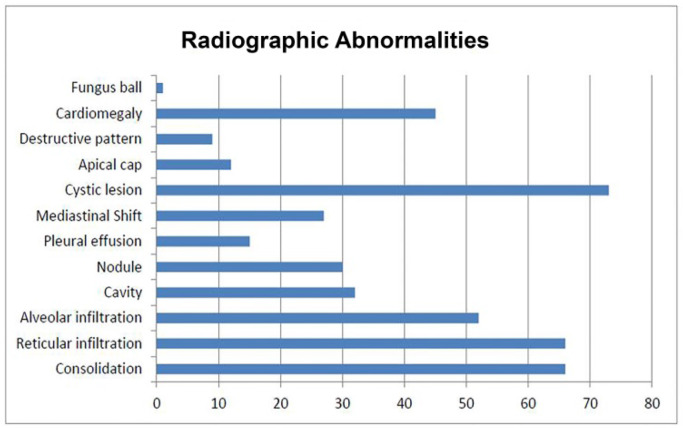
Radiographic findings in patients with hemoptysis

**Figure 3.**
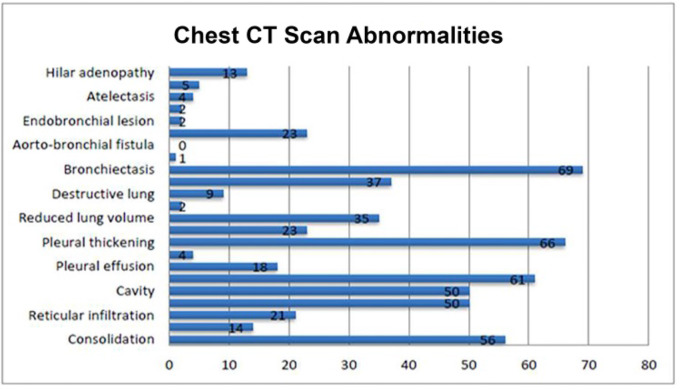
CT scan findings in patients with hemoptysis

As shown in Figure 2 the cystic lesions (42.1%), consolidation (37.9%), and reticular infiltration (37.9%) were the most common findings on plain CXR. On the other side, bronchiectasis and pleural thickening were the most common abnormalities on Lung CT scan (Figure 3).

**Figure 4.**
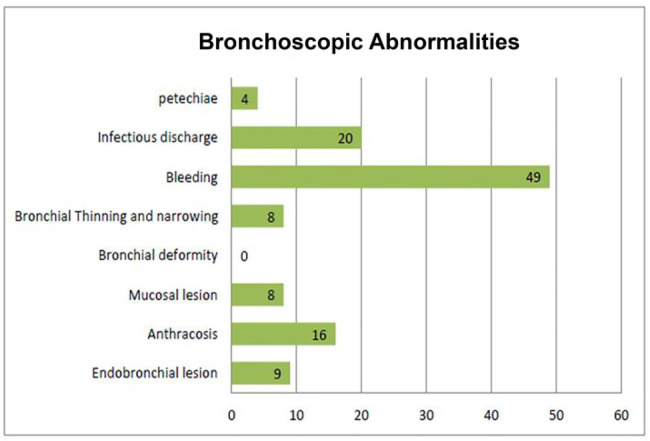
Bronchoscopic findings in patients with hemoptysis

**Figure 5.**
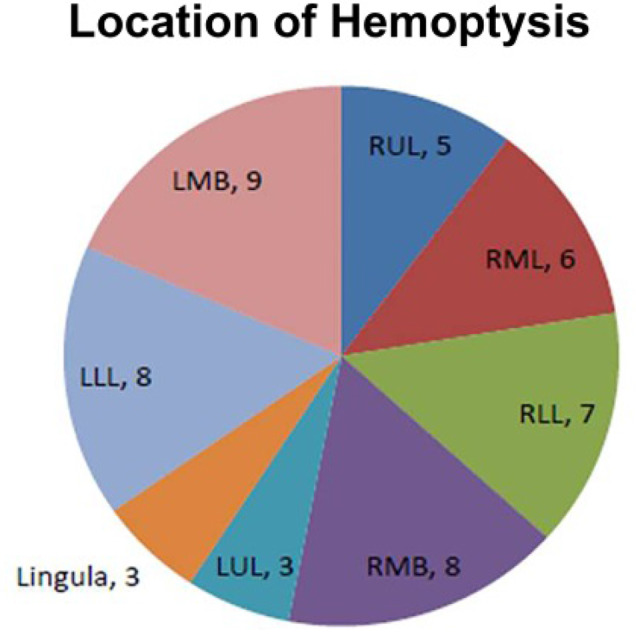
Location of hemoptysis in patients

Bronchoscopic findings and bleeding site are classified in Figures 4 and 5, respectively. The main finding was bleeding (Figure 4). During BA angiography, shunting to PA or PV, parenchymal blush, tortuosity and enlargement, aneurismal lesion, and contrast extravasations were detected in 80 (42.4%), 54 (28.5%), 167 (88.5%), 31 (16.4%), and 34 cases (18.2%), respectively. Among patients, 20 cases (10.7%) had no involved vessel. But 86(45.5%), 60(31.6%), and 23(12.3%) patients had 1, 2, and more than 2 involved vessels, respectively. The time interval with previous angioembolization was less than 3 weeks, 1 week to 1 month, 1–12 months, and 1–2 years in 1.8, 1.8, 7.2, and 3%, respectively.

We did not experience any major complication. Temporary chest pain, subintimal dissection, temporary dysphagia, and pancreatitis were seen in 12.3, 2.4, 5.1, and 0.5%, respectively. As explained before, we had lost 2 patients during index hospitalization. Both had history of complex cyanotic congenital heart diseases. The hemoptysis ablation was quite successful and bleeding had stopped promptly after the procedure. However, both patients died of severe right sided heart failure despite extensive support therapy.

## DISCUSSION

Traditionally surgery is considered as the ultimate therapy for hemoptysis. However, it is accompanied by high morbidity and mortality and the success rate is limited (9). Bronchial angioembolization is an emerging therapy, which allows a rapid and selective control of bleeding and with least morbidity and mortality(10,11). In the present study we have reported our result of bronchial angioembolization in 189 patients that referred to two large cardiovascular centers in Iran.

In our study, the main cause of massive hemoptysis was bronchiectasis followed by tuberculosis, aspergillus pneumonia and pulmonary hypertension, while worldwide, malignancy is the main reported etiology (6–8).

During our study we reached a success rate of 97.3% and recurrence rate of 28.3 % which is quite acceptable compared to global experience (12–20). In a systematic review performed by Panda et al (12), BAE was effective in 70 to 99 percent with a recurrence rate from 10 to 57 percent in various studies. Mainly the etiology of hemoptysis and the skill of the operator have influenced the effectiveness and the rate of recurrence. Ittrich et al. in their narrative review of BAE, have emphasized the operator’s experience and how the “profound knowledge of bronchial anatomy” was related to procedural success (15). Flight et al. in their investigation on 27 patients diagnosed with cystic fibrosis referring with massive hemoptysis showed that BAE is an effective procedure but with high risk of recurrence, showing perfectly the importance of etiology (20). An important factor was the extensiveness of hemoptysis ablation during the initial episode (12). Interestingly, the embolization agent might also affect the recurrence rate. In the present study, PVA and EmboSphere were the main agents used for BAE. Yoo et al. in their report of 108 patients that referred with major hemoptysis, tested the safety and efficacy of N-butyl cyanoacrylate. Their recurrence rate was 3.2% during five year follow up.

In our study, we did not encounter any major complication. Panda et al. (12) in their systematic review have reported a mean major complication rate of 0.1% with a range of 0 to 6.6%. Some of devastating major complications observed in this study included vascular perforation, mediastinal structure infarction, bronchoesophageal fistula, ischemic colitis and neurological complication such as transverse myelitis, cortical blindness and stroke. Temporary chest pain, subintimal dissection, temporary dysphagia, and pancreatitis were among our minor complications which have also been reported by other investigators (17, 18).

Ghanaati et al. (19) have also evaluated the role of BAE in Iranian population. Their case series of 30 patients showed that tuberculosis, bronchiectasis and lung cancer were the most common etiologies in their studied population. BAE was successful in all patients and no major complication has been encountered (19).

In conclusion, our study showed that bronchial angioembolization is considered a safe and effective approach in the treatment of patients with massive hemoptysis. The procedure success has a close relation with operator’s skill, bronchial anatomy and hemoptysis etiology. BAE might be considered an acceptable alternative therapy compared to surgery. However, further studies with larger sample size are required to attain more definite results.

## References

[1] Ittrich H Bockhorn M Klose H Simon M The Diagnosis and Treatment of Hemoptysis. Dtsch Arztebl Int 2017;114(21):371–81. 28625277 10.3238/arztebl.2017.0371 PMC5478790

[2] Cordovilla R Bollo de Miguel E Nuñez Ares A Cosano Povedano FJ Herráez Ortega I Jiménez Merchán R Diagnosis and Treatment of Hemoptysis. Arch Bronconeumol 2016;52(7):368–77. 26873518 10.1016/j.arbres.2015.12.002

[3] Bidwell JL Pachner RW Hemoptysis: diagnosis and management. Am Fam Physician 2005;72(7):1253–60. 16225028

[4] Bhalla A Pannu AK Suri V Etiology and outcome of moderate-to-massive hemoptysis: Experience from a tertiary care center of North India. Int J Mycobacteriol 2017;6(3):307–10. 28776532 10.4103/ijmy.ijmy_54_17

[5] Earwood JS Thompson TD Hemoptysis: evaluation and management. Am Fam Physician 2015;91(4):243–9. 25955625

[6] Jean-Baptiste E Clinical assessment and management of massive hemoptysis. Crit Care Med 2000;28(5):1642–7. 10834728 10.1097/00003246-200005000-00066

[7] Larici AR Franchi P Occhipinti M Contegiacomo A del Ciello A Calandriello L Diagnosis and management of hemoptysis. Diagn Interv Radiol 2014;20(4):299–309. 24808437 10.5152/dir.2014.13426 PMC4463269

[8] Tsoumakidou M Chrysofakis G Tsiligianni I Maltezakis G Siafakas NM Tzanakis N A prospective analysis of 184 hemoptysis cases: diagnostic impact of chest X-ray, computed tomography, bronchoscopy. Respiration 2006;73(6):808–14. 16446530 10.1159/000091189

[9] Velly JF Jougon J Laurent FS Valat P Massive haemoptysis: management and treatment. What is the role of surgery?. Rev Mal Respir 2005;22(5 Pt 1):777–84. 16272980 10.1016/s0761-8425(05)85635-9

[10] Radchenko C Alraiyes AH Shojaee S A systematic approach to the management of massive hemoptysis. J Thorac Dis 2017;9(Suppl 10):S1069–S1086. 29214066 10.21037/jtd.2017.06.41 PMC5696556

[11] Reechaipichitkul W Latong S Etiology and treatment outcomes of massive hemoptysis. Southeast Asian J Trop Med Public Health 2005;36(2):474–80. 15916059

[12] Panda A Bhalla AS Goyal A Bronchial artery embolization in hemoptysis: a systematic review. Diagn Interv Radiol 2017;23(4):307–17. 28703105 10.5152/dir.2017.16454 PMC5508955

[13] Yoo DH Yoon CJ Kang SG Burke CT Lee JH Lee CT Bronchial and nonbronchial systemic artery embolization in patients with major hemoptysis: safety and efficacy of N-butyl cyanoacrylate. AJR Am J Roentgenol. 2011;196(2):W199–204. 21257863 10.2214/AJR.10.4763

[14] Loh GA Lettieri CJ Shah AA Bronchial arterial embolisation for massive haemoptysis in cavitary sarcoidosis. BMJ Case Rep. 2013 J;2013. pii: bcr2012008268. 10.1136/bcr-2012-008268 PMC3603832 23355590

[15] Ittrich H Klose H Adam G Radiologic management of haemoptysis: diagnostic and interventional bronchial arterial embolisation. Rofo 2015;187(4):248–59. 25372159 10.1055/s-0034-1385457

[16] Mehta AS Ahmed O Jilani D Zangan S Lorenz J Funaki B Bronchial artery embolization for malignant hemoptysis: a single institutional experience. J Thorac Dis 2015;7(8):1406–13. 26380767 10.3978/j.issn.2072-1439.2015.07.39 PMC4561266

[17] Shao H Wu J Wu Q Sun X Li L Xing Z Bronchial artery embolization for hemoptysis: a retros pective observational study of 344 patients. Chin Med J (Engl) 2015;128(1):58–62. 25563314 10.4103/0366-6999.147811 PMC4837820

[18] Bhalla A Kandasamy D Veedu P Mohan A Gamanagatti S A retrospective analysis of 334 cases of hemoptysis treated by bronchial artery embolization. Oman Med J 2015;30(2):119–28. 25960838 10.5001/omj.2015.26 PMC4412455

[19] Ghanaati H Shakouri Rad A Firouznia K Jalali AH Bronchial artery embolization in life-threatening massive hemoptysis Iran Red Crescent Med J 2013;15(12):e16618. 24693401 10.5812/ircmj.16618 PMC3955516

[20] Flight WG Barry PJ Bright -Thomas RJ Butterfield S Ashleigh R Jones AM Outcomes Following Bronchial Artery Embolisation for Haemoptysis in Cystic Fibrosis. Cardiovasc Intervent Radiol 2017;40(8):1164–8. 28289842 10.1007/s00270-017-1626-0

